# A Functional Link Between Bir1 and the *Saccharomyces cerevisiae* Ctf19 Kinetochore Complex Revealed Through Quantitative Fitness Analysis

**DOI:** 10.1534/g3.117.300089

**Published:** 2017-07-28

**Authors:** Vasso Makrantoni, Adam Ciesiolka, Conor Lawless, Josefin Fernius, Adele Marston, David Lydall, Michael J. R. Stark

**Affiliations:** * Centre for Gene Regulation and Expression, University of Dundee, DD1 5EH, UK; † Wellcome Trust Centre for Cell Biology, University of Edinburgh, EH9 3BF, UK; ‡ Institute for Cell and Molecular Biosciences, Newcastle University, NE2 4HH, UK

**Keywords:** Bir1, Chromosome biorientation, Kinetochore, Iml3-Chl4 complex, yeast

## Abstract

The chromosomal passenger complex (CPC) is a key regulator of eukaryotic cell division, consisting of the protein kinase Aurora B/Ipl1 in association with its activator (INCENP/Sli15) and two additional proteins (Survivin/Bir1 and Borealin/Nbl1). Here, we report a genome-wide genetic interaction screen in *Saccharomyces cerevisiae* using the *bir1-17* mutant, identifying through quantitative fitness analysis deletion mutations that act as enhancers and suppressors. Gene knockouts affecting the Ctf19 kinetochore complex were identified as the strongest enhancers of *bir1-17*, while mutations affecting the large ribosomal subunit or the mRNA nonsense-mediated decay pathway caused strong phenotypic suppression. Thus, cells lacking a functional Ctf19 complex become highly dependent on Bir1 function and vice versa. The negative genetic interaction profiles of *bir1-17* and the cohesin mutant *mcd1-1* showed considerable overlap, underlining the strong functional connection between sister chromatid cohesion and chromosome biorientation. Loss of some Ctf19 components, such as Iml3 or Chl4, impacted differentially on *bir1-17* compared with mutations affecting other CPC components: despite the synthetic lethality shown by either *iml3*∆ or *chl4*∆ in combination with *bir1-17*, neither gene knockout showed any genetic interaction with either *ipl1-321* or *sli15-3*. Our data therefore imply a specific functional connection between the Ctf19 complex and Bir1 that is not shared with Ipl1.

To maintain genomic integrity, it is essential that every chromosome be faithfully transmitted to both progeny during cell division. Genomic instability is a characteristic of cancer cells, and chromosome number alterations (aneuploidy) caused by gain or loss of chromosomes are thought to be one of the driving forces behind tumor progression (Hanahan and Weinberg 2011). To help ensure accurate chromosome segregation, sister chromatids generated by DNA replication are held together by protein complexes termed cohesin. The sister kinetochores, multiprotein complexes assembled at sister centromeres to mediate their attachment to microtubules (Lampert and Westermann 2011; Santaguida and Musacchio 2009), become linked to microtubules emanating from opposite spindle poles as they align on the mitotic spindle during metaphase (Nasmyth and Haering 2009). This state of attachment (chromosome biorientation) ensures that when cohesin is removed as cells enter anaphase, sister chromatids are pulled in opposite directions and each daughter receives exactly one copy of each chromosome (Tanaka *et al.* 2005).

Aurora B protein kinase has emerged over the past 15 yr as a key regulator promoting chromosome biorientation (Tanaka *et al.* 2005). Although there is an intrinsic bias favoring bioriented attachment of sister chromatids to the mitotic spindle that is most readily seen when the spindle pole bodies (SPBs) have already separated (Indjeian and Murray 2007; Verzijlbergen *et al.* 2014), achievement of biorientation is not automatic and attachment errors occur that would lead to chromosome mis-segregation if they were left uncorrected. Aurora B kinase corrects such errors, promoting detachment of incorrect attachments through phosphorylation of proteins at the kinetochore, such that correct attachments have a chance to replace them (Liu *et al.* 2009; Tanaka *et al.* 2002). Aurora B/Ipl1 kinase forms part of the chromosomal passenger complex (CPC; see Ruchaud *et al.* 2007) together with three other conserved proteins (yeast names in parentheses): INCENP (Sli15), Survivin (Bir1), and Borealin (Nbl1). INCENP, Survivin, and Borealin associate via a triple helical interaction (Jeyaprakash *et al.* 2007) and INCENP contains a domain that binds to and activates Aurora B kinase (Kang *et al.* 2001). Error correction by Ipl1 kinase is an essential process and yeast cells show massive mis-segregation of chromosomes during division in its absence (Biggins *et al.* 1999). In addition to the CPC, efficient chromosome biorientation also requires the accumulation of cohesin around the centromere in yeast (pericentromeric cohesin), as well as pericentromeric condensin and the protein Sgo1, which also interacts with this region of yeast chromosomes (Marston 2015). The contribution of pericentromeric cohesin, condensin, and Sgo1 may be to enforce a geometry that underlies the intrinsic bias toward chromosome biorientation (Verzijlbergen *et al.* 2014), while Sgo1 may be needed to sense when sister kinetochores are under tension from the mitotic spindle, thereby indicating that they are correctly bioriented (Marston 2015). The CPC-mediated error correction mechanism has generally been considered to involve inner centromere–localized Aurora B/Ipl1 (see Lampson and Cheeseman 2011). In yeast, CPC interaction with the inner centromere is targeted by Bir1 through its interactions with Ndc10 (Cho and Harrison 2012; Yoon and Carbon 1999) and with histone H2A phosphorylated on Ser-121 by Bub1 kinase (Kawashima *et al.* 2010). However, the importance of inner centromeric localization of the CPC has recently been called into question by the surprising finding that the Ipl1-Sli15 complex in yeast can still provide error correction, even in the absence of Bir1 or Nbl1, if it is delocalized from kinetochores by deletion of the first 228 residues of Sli15 that normally anchor Ipl1-Sli15 to Bir1 and Nbl1 (Campbell and Desai 2013; Fink *et al.* 2017; Jeyaprakash *et al.* 2007).

We previously generated a temperature-sensitive allele (*bir1-17*) supporting normal proliferation and chromosome biorientation at 26°, but which fails to proliferate and shows a chromosome biorientation defect at 37° (Makrantoni and Stark 2009). *bir1-17* contains 11 point alterations within the C-terminus half of the protein, seven of which are localized within the C-terminus 297 residues of Bir1 that can provide its essential function (Widlund *et al.* 2006). Five point alterations are within the C-terminus 228 residues of Bir1 that interact strongly with both Nbl1 and Sli15 (Nakajima *et al.* 2009), and two lie within residues 889–941 that correspond to a domain proposed to form the triple helical interaction that is conserved in the human CPC (Jeyaprakash *et al.* 2007; Nakajima *et al.* 2009). One of these (L924S) affects a hydrophobic residue that is directly involved in the triple helical interaction, and is also mutated in two other conditional *bir1* alleles (Shimogawa *et al.* 2009). Thus *bir1-17* is likely to affect the interaction of the mutant protein with the other CPC components, although we have not examined this directly. Some of the point mutations are also located within a region of Bir1 that is known to interact with Ndc10 (Thomas and Kaplan 2007).

To understand better the role of Bir1 and the proteins and processes with which it interacts, we carried out a genome-wide synthetic interaction screen using the *bir1-17* mutant. We found that the *bir1-17* mutant is strongly enhanced by mutations affecting components of the Ctf19 kinetochore complex, including Chl4 and Iml3, and in the W303 background both *chl4*∆ and *iml3*∆ are synthetic lethal with *bir1-17*. Surprisingly, the synthetic lethal interactions between *bir1-17* and either *chl4*∆ or *iml3*∆ are specific to *bir1-17* and were not seen with either *sli15-3* or *ipl1-321*, which each confer a much stronger Ts^−^ phenotype than *bir1-17* (Makrantoni and Stark 2009). The yeast Ctf19 complex is a group of inner kinetochore proteins that are analogous to the CCAN complex of metazoan kinetochores (Lampert and Westermann 2011; Santaguida and Musacchio 2009). Most yeast Ctf19 complex components are nonessential for proliferation, although gene knockouts confer elevated chromosome mis-segregation and reduced association of cohesin, condensin, Sgo1, and Ipl1 with centromere-proximal (pericentromeric) chromatin (Kiburz *et al.* 2005; Fernius and Marston 2009; Verzijlbergen *et al.* 2014). Our data therefore imply a specific functional connection between Bir1 and these two Ctf19 complex components that is critical for CPC function, and supports the notion that the *bir1-17* mutation affects CPC function in a fundamentally different way to *ipl1* mutations that simply reduce its ability to phosphorylate its targets. Our findings are consistent with the notion that delocalization of the CPC from kinetochores may make cells more dependent on mechanisms involving the Ctf19 complex that impart the intrinsic bias toward biorientation.

## Materials and Methods

### Yeast strains and general methods

Basic yeast methods, growth media, and routine recombinant DNA methodology were performed as previously described (Amberg *et al.* 2005; Gietz *et al.* 1992). Unless stated otherwise, all yeast strains used in this study (Table 1) are derivatives of W303-1a (Thomas and Rothstein 1989) and have the following markers: *ade2-1 his3-11*, *15 leu2-3*, *112 trp1-1 ura3-1 can1-100 ssd1-d2* Gal^+^. However, synthetic interactions screening was performed as previously described, using the BY strain background for reasons of strain compatibility with the genome-wide gene knockout collection (Addinall *et al.* 2011). To verify genetic interactions detected in the BY background, deletion strains were made W303 background by using the pFA6a-HIS3MX6 cassette as previously described (Longtine *et al.* 1998), and then crossed with *bir1-17* in the same background. Deletion of *IRC15* was performed such that the last eight codons of *CTF19* (which overlap with *IRC15*) were retained.

**Table 1:** Yeast strains

Strain*^a^*	Genotype	Source
AM1145	*MAT***a** *MCD1-6HA*	Fernius and Marston (2009)
AM1176	*MAT***a**	Fernius and Marston (2009)
AM3442	*MAT****a*** *MCD1-6HA chl4*Δ::*KanMX6*	Fernius and Marston (2009)
AM9332	*MAT***a** *MCD1-6HA bir1-17*::*NatMX*	This study
AM14933	*MAT*α *sli15*∆*2-228*	This study
Deletion collection*^b^*	*MAT***a** *his3*∆*1 leu2*∆*0 met15*∆*0 ura3*∆*0 yfg*∆::*KanMX4*	Winzeler *et al.* (1999)
K699	*MAT***a**	Kim Nasmyth
DLY4242*^b^*	*MAT*α *can1*∆::*STE2pr-Sphis5 lyp1*∆ *his3*∆*1 leu2*∆*0 ura3*::*NatMX met15*∆*0*	Charles Boone strain Y8835
T1654	*MAT*α *ipl1-321*	Tomo Tanaka
T1812	*MAT****a*** *ipl1-2*	Tomo Tanaka
T1819	*MAT***α** *sli15-3*	Tomo Tanaka
VMY26	*MAT***a** *bir1-17*::*NatMX*	This study
VMY165*^b^*	*MAT*α *can1*∆::*STE2pr-Sphis5 lyp1*∆ *his3*∆*1 leu2*∆*0 ura3*∆*0 met15*∆*0 HphMX6*::*BIR1*	This study
VMY179*^b^*	*MAT*α *can1*∆::*STE2pr-Sphis5 lyp1*∆ *his3*∆*1 leu2*∆*0 ura3*∆*0 met15*∆*0 HphMX6*::*bir1-17*::*NatMX*	This study
VMY199	*MATa iml3*Δ::*HIS3MX6*	This study
VMY206	*MATa chl4*Δ::*HIS3MX6*	This study
VMY229	*MAT****a****/MAT***α** *IML3/iml3Δ*::*HIS3 BIR1/bir1-17*::*NatMX*	This study
VMY261	*MAT****a*** *TOR1-1 fpr1*::*loxP-LEU2-loxP RPL13A*-2**×***FKBP12*::*loxP-TRP1-loxP IML3-FRB*::*HIS3MX6*	This study
VMY262	*MAT****a*** *TOR1-1 fpr1*::*loxP-LEU2-loxP RPL13A*-2**×***FKBP12*::*loxP-TRP1-loxP CHL4-FRB*::*HIS3MX6*	This study
VMY263	*MAT****a*** *ipl1-321 chl4*Δ::*HIS3MX6*	This study
VMY265	*MAT****a*** *ipl1-321 iml3*Δ::*HIS3MX6*	This study
VMY269	*MAT****a****/MAT***α** *CHL4/chl4Δ*::*HIS3MX6 BIR1/bir1-17*::*NatMX*	This study
VMY302	*MAT****a*** *TOR1-1 fpr1*::*loxP-LEU2-loxP RPL13A*-2**×***FKBP12*::*loxP-TRP1-loxP IML3-GFP-FRB*::*HIS3MX6*	This study
VMY303	*MAT****a*** *TOR1-1 fpr1*::*loxP-LEU2-loxP RPL13A*-2**×***FKBP12*::*loxP-TRP1-loxP CHL4-GFP-FRB*::*HIS3MX6*	This study
VMY304	*MAT****a*** *TOR1-1 fpr1*::*loxP-LEU2-loxP RPL13A*-2**×***FKBP12*::*loxP-TRP1-loxP AME1-FRB*::*HIS3MX6*	This study
VMY305	*MAT****a*** *TOR1-1 fpr1*::*loxP-LEU2-loxP RPL13A*-2**×***FKBP12*::*loxP-TRP1-loxP OKP1-FRB*::*HIS3MX6*	This study
VMY328	*MAT****a*** *TOR1-1 fpr1*::*loxP-LEU2-loxP RPL13A*-2**×***FKBP12*::*loxP-TRP1-loxP IML3-FRB*::*HIS3MX6 bir1-17*::*NAT*	This study
VMY330	*MAT****a*** *TOR1-1 fpr1*::*loxP-LEU2-loxP RPL13A*-2**×***FKBP12*::*loxP-TRP1-loxP CHL4-FRB*::*HIS3MX6 bir1-17*::*NAT*	This study
VMY398	*MAT***a** *iml3*Δ::*HIS3MX6 ipl1-2*	This study
VMY399	*MAT***a** *chl4*Δ::*HIS3MX6 ipl1-2*	This study
VMY402	*MAT*α *iml3*Δ::*HIS3MX6 sli15-3*	This study
VMY405	*MAT***α** *chl4*Δ::*HIS3MX6 sli15-3*	This study
VMY406	*MAT***a** *chl4*Δ::*HIS3MX6 sli15*∆*2-228*	This study
VMY408	*MAT***a** *iml3*Δ::*HIS3MX6 sli15*∆*2-228*	This study
VMY410	*MAT***a** *TOR1-1 fpr1*::*loxP-LEU2-loxP RPL13A*-2**×***FKBP12*::*loxP-TRP1-loxP IML3-FRB*::*HIS3MX6 ura3*::*GAL-SGO1*::*URA3*	This study
VMY411	*MAT***a** *TOR1-1 fpr1*::*loxP-LEU2-loxP RPL13A*-2**×***FKBP12*::*loxP-TRP1-loxP CHL4-FRB*::*HIS3MX6 ura3*::GAL-SGO1::URA3	This study
VMY412	*MAT***a** *TOR1-1 fpr1*::*loxP-LEU2-loxP RPL13A*-2**×***FKBP12*::*loxP-TRP1-loxP IML3-FRB*::*HIS3MX6 bir1-17*::*NatMX ura3*::*GAL-SGO1*::*URA3*	This study
VMY413	*MAT***a** *TOR1-1 fpr1*::*loxP-LEU2-loxP RPL13A*-2**×***FKBP12*::*loxP-TRP1-loxP CHL4-FRB*::*HIS3MX6 bir1-17*::*NatMX ura3*::*GAL-SGO1*::*URA3*	This study
VMY416	*MAT***a** *nbl1-6*::*LEU2*	This study
VMY418	*MAT***a** *chl4*Δ::*HIS3MX6 nbl1-6*::*LEU2*	This study
VMY420	*MAT***a** *iml3*Δ::*HIS3MX6 nbl1-6*::*LEU2*	This study
Y7092*^b^*	*MAT*α *can1*∆::*STE2pr-Sphis5 lyp1*∆ *his3*∆*1 leu2*∆*0 ura3*∆*0 met15*∆*0*	This study

a All strains are W303 unless otherwise indicated, and contain *ade2-1 his3-11*,*15 leu2-3*,*112 trp1-1 ura3-1 can1-100 ssd1-d2*.

b S288C genetic background.

### Chromatin immunoprecipitation

Cohesin association with centromeric, pericentromeric, and arm sequences from chromosome IV was assessed in strains expressing HA-tagged Mcd1 using chromatin immunoprecipitation with anti-HA antibody (clone 12CA5) followed by qPCR analysis, performed as previously described (Fernius and Marston 2009; Fernius *et al.* 2013), using a Roche LightCycler and Express SYBR Green reagent (Invitrogen). PCR primers are listed in Supplemental Material Table S1.

### Quantitative fitness analysis using bir1-17

To generate a *bir1-17* strain in the S288C background suitable for synthetic gene array (SGA) screening, Y1082 was first transformed with a PCR fragment amplified from pFA6a-HpHMX6 (Hentges *et al.* 2005) using primers #536 and #537 (Table S1), such that the *Hph* marker replaced the region 236–297 bp upstream of the *BIR1* open reading frame (ORF), generating VMY165. This strain was next transformed with a PCR fragment made using primers #208 and #575 (Table S1) to amplify the *bir1-17*::*NatMX* construct from VMY26, in which the region 49-71 bases downstream of the *bir1-17* ORF was replaced by the *NatMX* marker from pAG25 (Goldstein and McCusker 1999). This generated VMY179, from which the *bir1-17* region was amplified and sequenced to verify presence of all of the base changes in *bir1-17* (Makrantoni and Stark 2009). Figure S1 shows the doubly marked *bir1-17* locus generated. Flanking *bir1-17* with two different markers and then selecting for both during SGA greatly reduced the possibility that *bir1-17* could be separated from its markers by recombination. VMY179 grew normally at temperatures below 37° but, while still clearly temperature-sensitive, showed some growth at 37°, particularly when arrayed by pinning.

SGA analysis by crossing VMY179 to the systematic yeast gene deletion collection was performed as already described (Addinall *et al.* 2008, 2011). Quantitative fitness analysis (QFA) of the double mutants to generate strain fitnesses and genetic interaction strengths (GIS) was performed as previously reported (Addinall *et al.* 2011), by screening at 20, 27, and 37°. Fitness and GIS calculations, including *t*-tests for the significance of GIS, were carried out and plots were generated using the QFA R package (version 0.0-43; http://qfa.r-forge.r-project.org/). Four replicate *bir1-17* strain (VMY179) crosses and eight replicate control strain DLY4242 (*ura3*::*NatMX*) crosses were analyzed. QFA data are summarized in Tables S2–S4 in File S2, following removal of all genes tightly linked to the query *bir1-17* mutation (*i.e.*, located within 20 kb of *bir1-17* on chromosome X) and a standard set of genes related to the genetic selections used in SGA that are therefore incompatible with SGA (*ARG82*, *ARG5,6*, *ARG4*, *ARG2*, *ARG3*, *ARG81*, *ARG80*, *ARG7*, *ARG1*, *ARG8*, *HIS7*, *HIS4*, *HIS2*, *HIS1*, *HIS6*, *HIS5*, *LEU2*, *LEU1*, *LEU5*, *LEU3*, *LEU4*, *LEU9*, *LYS2*, *LYS21*, *LYS20*, *LYS14*, *LYS4*, *LYS5*, *LYS12*, *LYS1*, *LYS9*, and *CCS1*). To identify potentially significant phenotypic enhancers and suppressors, double mutants with *q*-value [false discovery rate (FDR) corrected *p*-value ≤ 0.05] and either a negative GIS (enhancers) or positive GIS (suppressors) were selected (Tables S5–S8 in File S2). GO terms enriched in the enhancer and suppressor gene subsets were determined using the GO Term Finder (version 0.83; Boyle *et al.* 2004). as implemented by the *Saccharomyces* Genome Database (SGD; Cherry *et al.* 2012, queried December 2016) with a *p*-value cut-off of ≤0.01. All recognized *Saccharomyces cerevisiae* nuclear-encoded ORFs within the systematic deletion collection, but lacking the *bir1-17*–linked genes and the SGA-incompatible genes listed above, were used as the background set for determining GO term enrichment (4235 genes; the full list is shown in each of Table S2, Table S3, and Table S4 in File S2). For further analysis of strong negative genetic interactors (GIS ≤ −25) identified at 20 or 27°, growth of the individual control and *bir1-17* double mutants was examined. Where at least three out of four *bir1-17 yfg*∆ replicates failed to grow but at least six out of eight control *ura3*::*NatMX yfg*∆ replicates grew, then the high negative GIS was considered to represent a synthetic lethal interaction (*yfg*∆: your favorite gene deletion; used to indicate one of the ∼4200 viable yeast gene deletions from the systematic deletion collection). Fitness plots were generated from the QFA data using iRVis (http://qfa.r-forge.r-project.org/visTool/), which is a part of the QFA software package, with significant negative and positive genetic interactors (*i.e.*, *q*-values of ≤ 0.05 defined by *t*-test) colored blue and red, respectively.

### Data availability

Yeast strains are available on request. File S1 contains detailed descriptions of all supplemental files. File S2 contains Tables S2–S8. File S3 summarizes GO analysis of *bir1-1*7 enhancers and File S4 summarizes GO analysis of *bir1-17* suppressors. The authors state that all data necessary for confirming the conclusions presented in the article are represented fully within the article and the supplemental material.

## Results

### QFA identifies gene deletions that interact with bir1-17

To identify enhancers and suppressors of *bir1-17* that might indicate specific functional interactions, we used SGA technology to cross *bir1-17* to the collection of ∼4200 viable systematic gene deletion strains, followed by QFA (Addinall *et al.* 2011) to identify suppressing or enhancing genetic interactions. *bir1-17* was originally isolated in the W303 genetic background and confers a recessive, temperature-sensitive growth defect that is clearly evident at 37° (Makrantoni and Stark 2009). To investigate the best approach for identifying *bir1-17* suppressors and enhancers, we first compared growth of the S288C background control and *bir1-17* strains after arraying by either pinning or spotting. Figure S2 shows that when strains were arrayed by pinning, the fitness defect of *bir1-17* was hard to detect even at higher incubation temperatures (Figure S2A). In contrast, when dilutions of the two strains were arrayed by spotting onto the screening plates then the fitness defect of *bir1-17* was readily detectable at 37° (Figure S2B). We therefore carried out QFA analysis by spotting rather than pinning the arrays of control and *bir1-17* double mutants generated by SGA.

Double *bir1-17 yfg*∆ mutants were generated at 23°. QFA was subsequently performed by spotting out and screening growth at 20, 27, and 37°, calculating the GIS and *q*-value (FDR-corrected *p*-value) for each double mutant to indicate the magnitude of the genetic interaction between *bir1-17* and each *yfg*∆ gene knockout and its statistical significance, respectively (Tables S2–S4 in File S2; data ranked in ascending order of GIS, starting with the most negative interactions). Since *bir1-17* cells spotted at 37° show a clear growth defect, we chose to focus primarily on this dataset. A *q*-value threshold of ≤0.05 was applied to identify a subset of statistically significant genetic interactions at each screening temperature. QFA data were summarized in the form of fitness plots, in which the fitness of each *bir1-17 yfg*∆ strain is plotted against the fitness of the corresponding control *ura3*∆ *yfg*∆ strain.

**Figure 1:**
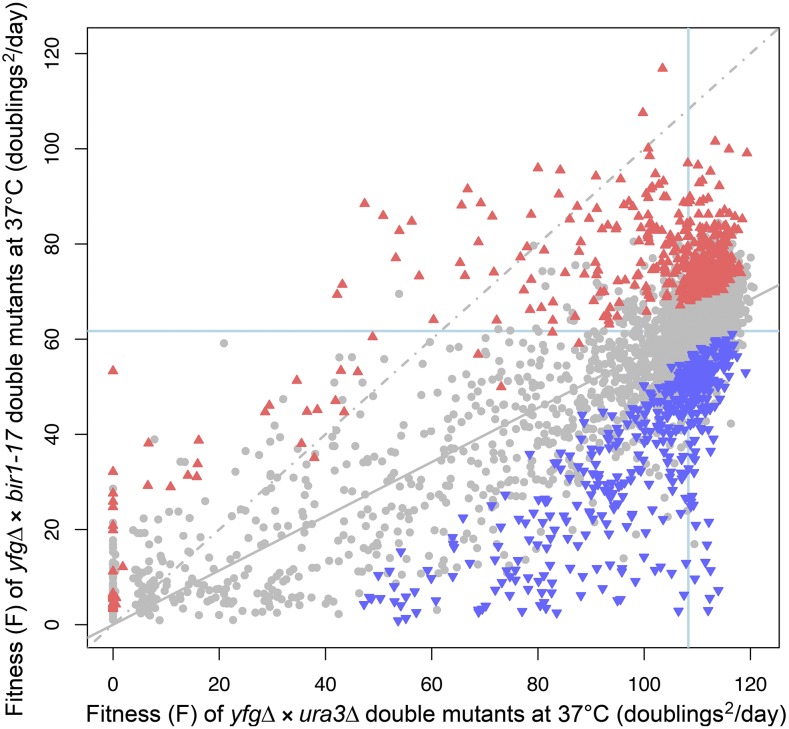
Fitness plot of *bir1-17* double mutants at 37°. Following four replicate crosses of *bir1-17* with the yeast genome knockout collection, quantitative fitness analysis of each *bir1-17 yfg*∆ (“your favorite gene deletion”) strain was carried out at 37° and mean fitness plotted against the mean fitness observed from eight replicates of a control cross between a *ura3*∆ strain and the knockout collection. Gene deletions that significantly enhanced (blue triangles) or suppressed (red triangles) the growth defect of a *bir1-17* strain are indicated, with all other nonsignificant deletions indicated as gray circles. A significant interaction was defined as one with a *q*-value (FDR-corrected *p*-value; see Addinall *et al.* 2011) ≤ 0.05, with enhancers having a negative genetic interaction strength (GIS) and suppressors having a positive GIS. The line of equal growth (gray dashed) and a population model of expected fitness under the assumption of genetic independence (solid gray; a regression line based on all the data points) are also indicated. The blue lines show the average position of *his3*∆ strains as a proxy for wild-type growth.

Figure 1 shows the fitness plot for *bir1-17 yfg*∆ mutants screened at 37°, indicating all statistically significant enhancers (negative GIS; blue triangles) and suppressors (positive GIS; red triangles). Table S5 and Table S8 in File S2 list the statistically significant *bir1-17* enhancers and suppressors, respectively, identified by screening at 37°, while Table S6 and Table S7 in File S2 present the statistically significant enhancers at 20 and 27° for comparison. In the fitness plot, the dashed gray line indicates where points should lie when deletion mutants show identical fitness in combination with either *bir1-17* or the control *ura3*∆ mutation (the line of equal growth). The regression line of the actual data points is indicated by the solid line. Downward displacement of this regression line away from the line of equal growth (Figure 1) is consistent with the temperature-sensitive phenotype of *bir1-17* that was clearly evident following spotting out under QFA conditions (Figure S2), and is in contrast to the fitness plots from the 20 and 27° screens (Figure S3).

To look in an unbiased manner for relationships between genes identified as statistically significant enhancers and suppressors of *bir1-17* at 37°, we searched for GO terms within the process, function, and component ontologies that showed significant enrichment in the enhancers and suppressors. We focused primarily on strong interactions (|GIS| ≥ 25; mapped onto the 37° fitness plot in Figure S4), but also searched using enhancers and suppressors with |GIS| ≥ 10 for comparison. These data are summarized in File S3 (enhancers) and File S4 (suppressors). We also manually examined the position on the 37° fitness plot of the members of each of the core protein complexes defined by Benschop *et al.* (2010), as a means of identifying consistent patterns of genetic interaction with *bir1-17* that might reflect functional interactions between Bir1 and specific cellular processes. Table 2 summarizes the proportion of *bir1-17* enhancers or suppressors identified at each screening temperature. Around 3% of *yfg*∆ knockouts strongly enhanced *bir1-17* in the 37° screen (GIS ≤ −25), while 2.2% of knockouts strongly suppressed (GIS ≥ 25). This was in contrast to the 20 and 27° screens, where the proportion of strong interactions was far less.

**Table 2:** Summary of *bir1-17* suppressors and enhancers

	Enhancers (%)*^a^*			Suppressors (%)*^a^*		
QFA Screening Temperature	All	GIS < −10	GIS < −25	All	GIS > 10	GIS > 25
37°	11.1	8.5	2.9	11.3	8.0	2.2
27°	2.5	2.1	0.3	2.1	1.3	0.1
20°	1.7	1.3	0.2	1.6	0.5	0.1

a Based on SGA using 4235 gene deletions and requiring a *q*-value of < 0.05.

### Phenotypic enhancers of bir1-17

It was quickly apparent from looking at the *bir1-17* enhancers identified at 37° (Table S5 in File S2) that knockouts of almost any of the nonessential components of the Ctf19 kinetochore complex (*CHL4*, *CTF3*, *CTF19*, *IML3*, *IRC15*, *MCM16*, *MCM21*, *MCM22*, and *NKP1*; see Biggins 2013) strongly enhanced *bir1-17*; only *NKP2* was not identified as an enhancer. *IRC15* is included here because of the overlap of its full-length deletion with *CTF19*, as discussed below. All of these knockouts except *nkp1*∆ fell into the strong enhancer category (*i.e.*, GIS ≤ −25), and five out of the six strongest enhancers were members of the Ctf19 complex (Table S5 in File S2). Only *MCM21* and *CTF19*, encoding the two nonessential members of the COMA subcomplex (Ame1 and Okp1 are both essential; De Wulf *et al.* 2003; Ortiz *et al.* 1999), were among the strongest enhancer of *bir1-17* at all three screening temperatures. The other members of the Ctf19 complex were either weaker enhancers at 20° and 27° than at 37°, or in some cases, not identified as significant enhancers at all. It is striking that in contrast to the two lower temperature screens, the 37° screen placed knockouts of genes encoding all the Ctf19 complex components except *NKP1* and *NKP2* in a tight cluster at the lower right of the plot, indicating that the knockouts had little or no defect in the control (*ura3*∆) background, but in contrast had a very strong defect when combined with *bir1-17* (see Figure S5). Thus, screening at 37° when double mutants are close to the *bir1-17* maximum permissive temperature greatly assisted in identifying genetic interactions between the core components of the Ctf19 complex and *bir1-17*.

GO analysis of the strong *bir1-17* enhancers (GIS ≤ −25) identified at 37° (see Table S5 in File S2) highlighted a number of specific GO terms concerned with chromosome segregation, sister chromatid cohesion, and microtubule-based processes, as well as more general terms including nuclear division and chromosome organization that encompassed many of the strongest negative genetic interactions (File S3). In addition to the Ctf19 complex, this analysis highlighted genes encoding all the *S. cerevisiae* kinesin-like proteins except *KIP1* (*i.e.*, *KIP2*, *KIP3*, *KAR3*, and *CIN8*), *VIK1* and *CIK1* (encoding Kar3-associated proteins; Manning *et al.* 1999), *BIK1* (a plus-end tracking protein related to CLIP-170; Berlin *et al.* 1990; Blake-Hodek *et al.* 2010), *TUB3* (α-tubulin), *CIN1* (encoding a β-tubulin folding factor; Hoyt *et al.* 1990, 1997) and *KAR9* (encoding a spindle positioning factor; Beach *et al.* 2000; Miller *et al.* 2000), although *kar9*∆ was inviable at 20 and 37° but was synthetic lethal with *bir1-17* at 27°. *bim1*∆, removing another plus-end tracking protein that binds to Kar9 and is also involved in spindle positioning (Beach *et al.* 2000; Miller *et al.* 2000), was also clearly a strong *bir1-17* enhancer at the two lower temperatures, although it fell outside our statistical significance cut-off at 37°. Thus cells lacking proper Bir1 function appear to become particularly dependent on the normal functioning of kinesins and microtubules.

GO analysis, together with consideration of the core protein complexes defined by Benschop *et al.* (2010), also identified knockouts of genes encoding any of the three members of the Ctf8/Ctf18/Dcc1 complex that is related to replication factor C (RFC^Ctf18^) as enhancers of *bir1-17* (although *ctf18*∆ fell just outside our cut-offs of *q* ≤ 0.05 and GIS ≤ −25 for significant strong enhancers). All three members of the Tof1/Mrc1/Csm3 complex that acts at stalled replication forks to promote sister chromatid cohesion (Bando *et al.* 2009; Tourriere *et al.* 2005; Xu *et al.* 2004) were also highlighted (although *tof1*∆ fell just outside our *q*-value cut-off). Like the Tof1 complex, the Ctf8 complex is also involved in sister chromatid cohesion (Mayer *et al.* 2001, 2004), highlighting the link between this process and CPC-dependent error correction. Examination of the core protein complexes also revealed negative enhancement of *bir1-17* by those viable knockouts in the systematic collection that affected RFC^Rad24^ (*RAD24*), RFC^Elg1^ (*ELG1*), a PCNA-like clamp (*RAD17* and possibly *DDC1*) and DNA polymerase epsilon [*DPB3*, *YBR277c* (::*DPB3*), and *DPB4* if the *q*-value cut-off is relaxed].

Broadening the GO analysis to consider enhancers with a GIS ≤ −10 did not change this overall picture but gave greater emphasis to some categories such as genes involved in chromatin modification (components of the Set3C, ISW, Compass, Ada, and Rpd3S complexes), histone exchange (*SWC5*, *VPS71*, and *VPS72*), tRNA wobble uridine modification (components of Elongator, *ATS1*, *KTI12*, *URM1*, *UBA4*, *NCS2*, *NCS6*, and SAP190), iron transport (*FET3* and *FTR1*), and peroxisomal function. Multiple components in each of these categories were strong, significant enhancers of *bir1-17* at 37° (Table S5 in File S2). While a potential functional connection between Bir1 and either iron transport or peroxisomes is not at all obvious, many of these other enhancers may function indirectly by altering the pattern of expression of proteins that have a direct functional relationship with Bir1. Other notable strong enhancers not falling into any of the above-mentioned categories included knockouts of the CENP-T-related kinetochore component *CNN1* that interacts with the Ndc80 kinetochore subcomplex (Bock *et al.* 2012; Malvezzi *et al.* 2013) and the *CPR6* peptidyl-prolyl *cis*-trans isomerase and its interacting protein encoded by *STI1* (Mayr *et al.* 2000). Figure S6 shows these groups of genes mapped onto the 37° fitness plot as an indicator of how consistently they interacted with *bir1-17* in the screen.

### Phenotypic suppressors of bir1-17

QFA carried out at 37° identified many potential suppressors of *bir1-17* that caused relief of the temperature-sensitive growth defect, including 94 strong suppressors (GIS ≥ 25; Table S8 in File S2). GO analysis of these genes (File S4) revealed highly significant enrichment for genes encoding components of the large ribosomal subunit (LRSU) or proteins involved in its rRNA processing and assembly, as well as for genes involved in mRNA catabolism, and in particular nonsense-mediated mRNA decay (NMD). This was further supported by our analysis of the core protein complexes of Benschop *et al.* (2010). Thus four out of the six strongest suppressors and almost one-third of all the strong (GIS ≥ 25) suppressors could be assigned roles either in ribosome biogenesis or as components of the ribosome. Strikingly, the vast majority of knockouts affecting the ribosome were specific for the LRSU: we isolated 27 *RPL* gene deletions (removing LRSU proteins) but only three *RPS* gene deletions (removing small ribosomal subunit proteins) as statistically significant *bir1-17* strong suppressors. Taking the unbiased approach of mapping all genes annotated as *RPL* and *RPS* in SGD onto the 37° fitness plot, we confirmed that genes in these two groups in general behaved very differently when knocked out and combined with *bir1-17* (Figure S7, compare A and B), while Figure S7C shows that the vast majority of all gene knockouts affecting either biogenesis of the LRSU or LRSU components caused phenotypic suppression of *bir1-17*.

Knockouts of the NMD genes *EBS1*, *NMD2*, *NAM7*, and *UPF3* were all within the strong suppressor set (*nam7*∆ was the second strongest suppressor), while the remaining components of the NMD pathway present in the systematic deletion collection (*DCN1* and *NMD4*) were also statistically significant suppressors but falling just below our GIS ≥ 25.0 cut-off (Table S8 in File S2). *SKI2* and *SKI3*, encoding two components of the Ski complex that are involved in a variety of RNA decay processes, including NMD and processes mediated by the exosome, were also identified. The remaining functionally related genes (*RRP6*, *SKI7*, *SKI8*, and YKL023w) were also suppressors falling slightly below our GIS cut-off. Figure S8A summarizes how the SKI, NMD, and exosome gene knockouts mapped onto the 37° fitness plot.

Although GO terms directly related to cell division were not specifically highlighted by our GO analysis even when suppressors with GIS ≥ 10 were included, one gene encoding a kinetochore component (*YBP2*) was found within the top 30 suppressors (File S4). Mapping the core protein complexes defined by Benschop *et al.* (2010) onto the 37° fitness plot also identified mutations affecting the COP9 signalosome as suppressors of *bir1-17*. The COP9 signalosome removes the NEDD8 homolog Rub1 from the yeast cullin Cdc53 and is required for cell cycle regulation at the G1/S boundary (Wee *et al.* 2002). Thus, both *csi1*∆ and *pci8*∆ were strong significant suppressors, while *csn9*∆, *rri1*∆, and *rri2*∆ were suppressors that fell just outside our GIS and/or *q*-value cut-offs (Figure S8B).

The basis for suppression by each of these classes of genes is not yet clear, but suppression by the first two groups of gene knockout is most likely related to alterations in the expression of proteins caused by changes in mRNA stability or alterations in the ribosome population. Since *bir1-17* is a temperature-sensitive loss of function mutant, it may be that these knockouts lead to elevated *Bir1-17* protein levels at higher temperatures that can compensate for the effect of the mutations it contains. In a previous QFA analysis, both NMD gene deletions and *RPL* (but not *RPS*) gene deletions were also found to suppress the temperature-sensitivity of a *cdc13-1* query mutation (Addinall *et al.* 2011). In this case, the NMD deletions were shown to operate through affecting levels of another protein (Stn1), which like Cdc13, is specifically involved in telomere function (Addinall *et al.* 2011). Conversely, both NMD and *RPL* gene deletions enhanced the phenotype of *yku70*∆ in a parallel QFA analysis (Addinall *et al.* 2011). It is therefore possible that loss of either the NMD pathway or specific *RPL* genes may lead to generalized effects on gene expression that can suppress or enhance specific mutations through effects on expression of functionally related genes.

### The genetic enhancement profiles of bir1-17 and ipl1-321 show both common and distinct features, many of which are shared with mcd1-1

**Figure 2:**
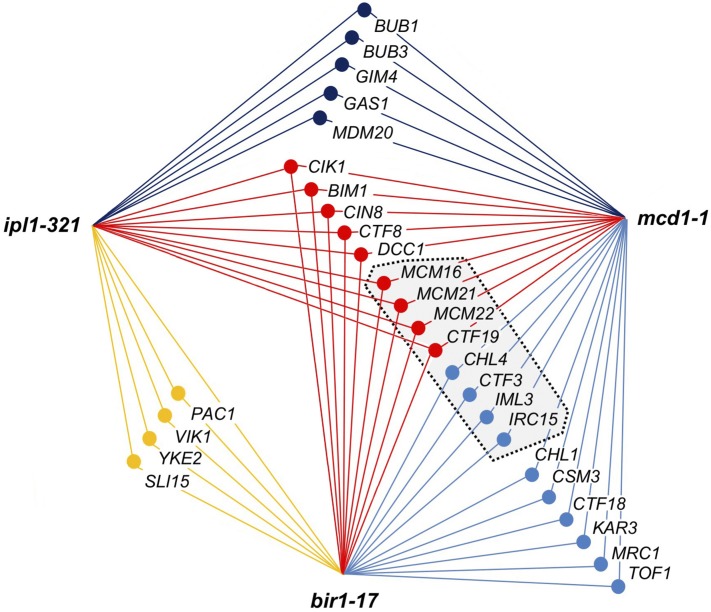
Comparison of the strong negative genetic interactors of *ipl1-321*, *bir1-17*, and *mcd1-1* revealed by SGA analysis. Genes showing synthetic lethality or strong negative genetic interaction with two or more of *ipl1-321*, *bir1-17*, or *mcd1-1* are connected to the relevant query mutations by lines that are color-coded according to the number of shared interactions as follows: yellow, negative genetic interactors shared by *ipl1-321* and *bir1-17*; dark blue, negative genetic interactors shared by *ipl1-321* and *mcd1-1*; light blue, negative genetic interactors shared by *bir1-17* and *mcd1-1*; and red, negative genetic interactors shared by all three query genes. The gray box with dashed outline encloses genes encoding components of the Ctf19 complex. Strong negative genetic interactors shown in the diagram were defined as follows: *ipl1-321*, all genes identified by Ng *et al.* (2009) as *ipl1-321* negative genetic interactors together with additional genes listed in SGD (Cherry *et al.* 2012, queried December 2016); *mcd1-1*, all genes identified by Ng *et al.* (2009) or present in the DRYGIN database (Koh *et al.* 2010, queried December 2016) as *mcd1-1* negative genetic interactors, together with additional genes listed in SGD (Cherry *et al.* 2012, queried December 2016); *bir1-17*, all strong negative enhancers identified in this work at any of the three screening temperatures that were shared with at least one of the other two mutations (*ipl1-321* or *mcd1-1*). Note that we find that deletion of *CTF19* is synthetic lethal with *ipl1-321* as shown (100% inviability of 25 *ctf19*::*KanMX ipl1-321* spores in 31 tetrads from a W303 background *ctf19*::*KanMX* × *ipl1-321* cross where single *ctf19*::*Kan*MX and *ipl1-321* segregants showed 100% and >97% viability, respectively). *IRC15* is included within the Ctf19 complex because its knockout also affects *CTF19*: see Confirmation of the negative genetic interactions between bir1-17 and the Ctf19, Ctf8-Ctf18-Dcc1 and Csm3-Mrc1-Tof1 complexes in W303 for details.

Since Ipl1 functions together with Sli15, Bir1, and Nbl1 within the yeast CPC complex, it might be expected that strains with loss-of-function mutations in each of these genes would show synthetic lethality or strong negative genetic interactions with a common set of nonessential gene knockouts that reflect their shared functional roles. Comprehensive analysis has yet to be performed with conditional alleles of either *sli15* or *nbl1*, but our study now enables comparison of the strong genetic enhancers of both *ipl1-321* and *bir1-17* to be made. Figure 2 shows that 13 of the strong genetic interactions of *bir1-17* established in our work are indeed shared with the known synthetic lethal interactions of *ipl1-321* (colored yellow and red). Overlap between strong negative genetic interactors of *ipl1-321* and those of the cohesin mutant *mcd1-1* (also called *scc1-73*) was noted previously (Ng *et al.* 2009) and is also the case here for *bir1-17*, with nine strong negative genetic interactions shared by all three mutant alleles (colored red in Figure 2). These data underline the strong functional links between sister chromatid cohesion, chromosome biorientation, and faithful sister chromatid segregation that operate during cell division.

Remarkably, the *ipl1-321* and *bir1-17* alleles each individually show at least as many strong negative genetic interactions with *mcd1-1* that they do not share with each other. For example, strains lacking several nonessential components of the Ctf19 complex that were identified in this study as the strongest negative enhancers of *bir1-17* are also synthetic lethal with, or strong enhancers of *mcd1-1*, but not *ipl1-321*. Conversely, both *ipl1-312* and *mcd1-1* show synthetic lethality with the spindle assembly checkpoint gene knockouts (*BUB1*, *BUB3*), whereas these mutations are only moderate negative genetic interactors of *bir1-17* at 27°, consistent with our earlier finding that *bir1-17* does not require a functional checkpoint for viability (Makrantoni and Stark 2009). The substantial differences between the profiles of *ipl1-321* and *bir1-17*, and in particular between those subsets of interactions that are shared with *mcd1-1*, support the notion that the *ipl1-321* and *bir1-17* mutations confer distinct effects on CPC function, although some of these differences could reflect either false positives or false negatives in one or another SGA screen. However, the apparent overlap between the strong enhancers of *bir1-17* and *mcd1-1* is nonetheless striking. We therefore next individually assessed a selection of the *bir1-17* genetic interactors identified in our screen both to verify the interactions and to address more systematically whether some interactions were really specific to *bir1-17* and not shared with *ipl1-321*. As a more robust approach to verification, we chose to do this in the W303 genetic background in which *ipl1-321* and *bir1-17* were both isolated (Biggins *et al.* 1999; Makrantoni and Stark 2009), and in which much of the work on CPC-mediated error correction in yeast has been carried out.

### Confirmation of the negative genetic interactions between bir1-17 and the Ctf19, Ctf8-Ctf18-Dcc1, and Csm3-Mrc1-Tof1 complexes in W303

To verify a selection of the interactions identified in the *bir1-17* QFA screen, we deleted a range of hits in the W303 background and carried out tetrad analysis following crosses with *bir1-17*. Table S9 summarizes those crosses where *bir1-17 yfg*∆ strains were found to be unconditionally lethal. In instances where such double mutants were viable, their relative fitness was examined by spotting out equivalent serial dilutions of strains on agar plates and assessing growth at different temperatures (Figure S9). Table S10 summarizes both sets of data and shows that, although some interactions could be readily verified in W303, other interactions could not.

**Figure 3:**
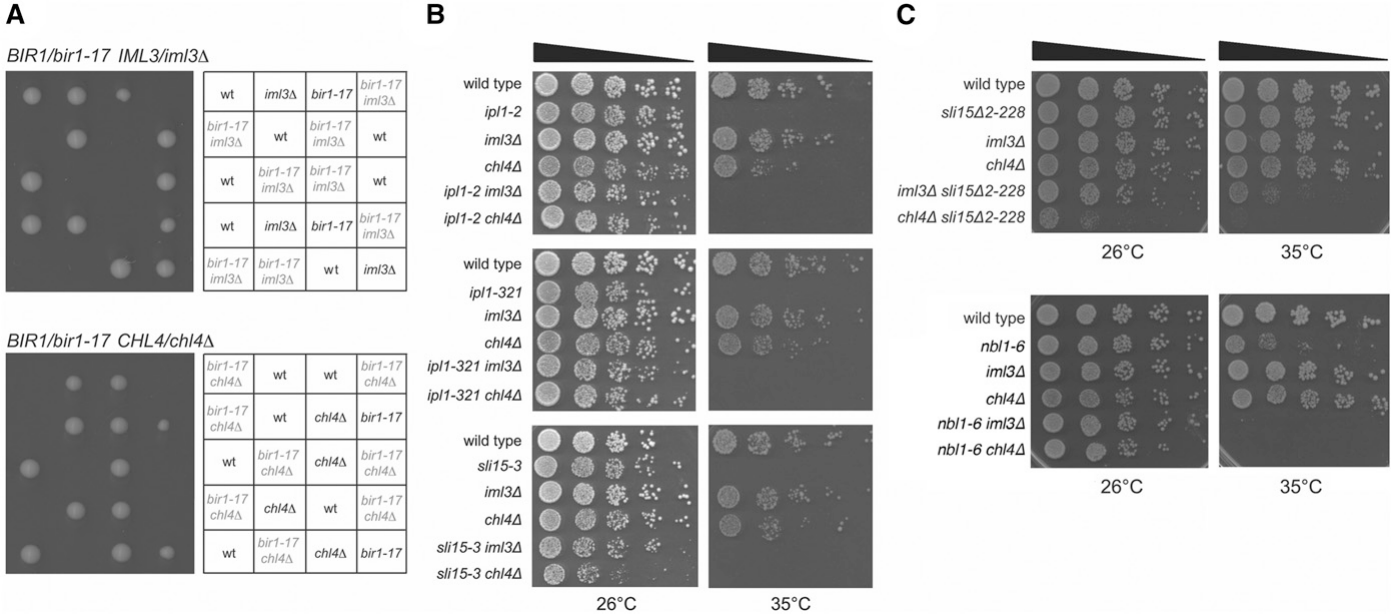
Genetic interactions of *iml3*∆ and *chl4*∆ with *bir1-17*, *ipl1*, and *sli15* mutations in the W303 genetic background. (A) *iml3*∆ and *chl4*∆ each show synthetic lethality with *bir1-17*. Progeny from five tetrads are shown, indicating the relevant genotypes of viable progeny and the deduced genotypes of inviable progeny. (B) *iml3*∆ and *chl4*∆ are viable when combined with *ipl1-2*, *ipl1-321*, *sli15-3*, and *sli15*∆*2-228*. Equivalent 10-fold dilutions of representative single and double mutants were grown at 26 or 35° for 2 d. Although the strong temperature-sensitive phenotype of *ipl1-2*, *ipl1-321*, and *sli15-3* is clearly evident at 35°, all double mutant combinations involving these alleles grew normally at 26°. (C) *iml3*∆ and *chl4*∆ are viable when combined with either *sli15*∆*2-228* or *nbl1-6* but show synthetic negative genetic interaction with both. While *iml3*∆ *sli15*∆*2-228* double mutants grew normally at 26°, *chl4*∆ *sli15*∆*2-228* grew poorly, and both *iml3*∆ *sli15*∆*2-228* and *chl4*∆ *sli15*∆*2-228* strains showed temperature sensitivity at 35° in comparison to the corresponding single mutant strains. *iml3*∆ *nbl1-6* and *chl4*∆ *nbl1-6* strains were also viable, but unlike the three individual mutant strains, were unable to grow at 35°.

A number of conclusions can be drawn from these data. First, all five knockouts of core Ctf19 complex components that we tested from within the group of strong *bir1-17* enhancers were synthetic lethal with *bir1-17* in the W303 background, as was the knockout of the one example of the kinesin group that we tested (*cin8*∆). *nkp1*∆ (identified as a weaker enhancer in our screen) showed no clear enhancement of *bir1-17* in W303, although deletion of its paralog *NKP2* caused strong enhancement of *bir1-17*. This verifies in W303 all five core Ctf19 complex components we identified by QFA as strong negative genetic interactors of *bir1-17*. Remarkably, the genetic interactions seen in W303 were actually stronger (synthetic lethality) compared with the QFA screen (strong enhancement). The unconditional lethality of either *iml3*∆ or *chl4*∆ with *bir1-17* in W303 (Figure 3A) is particularly striking in this regard, since the QFA screen only identified these knockouts as enhancers at 37° (see Tables S5–S7 in File S2). Although we identified *cnn1*∆ (deleting the yeast homolog of the kinetochore protein CENP-T) as a strong enhancer in the screen, this could not be reproduced in the W303 background. Second, negative genetic interactions between both the *Ctf8*-*Ctf18*-*Dcc1* complex (synthetic lethality with *dcc1*∆) and the *Csm3*-*Mrc1*-*Tof1* complex (negative genetic interaction with *mrc1*∆) could be confirmed in the W303 background, although the strong negative enhancement by loss of Csm3 seen by QFA could not be recapitulated. Finally, of the *bir1-17* enhancers identified that fell into the other functional groups discussed above, three could be verified in W303 as *bir1-17* enhancers (*iki3*∆, *vps71*∆, and *yku70*∆) but several others affecting the COMPASS (*bre2*∆, *spp1*∆), ISW (*chd1*∆) and Rpd3S (*sin3*∆) complexes showed little or no enhancement of *bir1-17* in W303.

Although we reproduced the negative genetic interaction between *bir1-17* and *irc15*∆ in W303 using a precise ORF knockout identical to that in the systematic collection used for the QFA screen (data not shown), this knockout also deletes the last eight sense codons of *CTF19*, with which *IRC15* overlaps on the opposite strand. Using a construct that removed the first 332 codons of the 499-codon *IRC15* ORF and thus left the *CTF19* ORF intact, we found no genetic interaction with *bir1-17* (Figure S9A), and so we conclude that *irc15*∆ was identified in our QFA screen because the systematic knockout interferes with Ctf19 function. It is also therefore likely that other mitotic phenotypes reported for *irc15*∆ mutants may result from effects on the overlapping *CTF19* gene rather than indicating functional consequences of Irc15 loss.

### bir1-17, but not ipl1-321, is dependent on the Iml3-Chl4 subcomplex for viability

Given that we identified several verifiable strong negative genetic interactions in our QFA analysis between *bir1-17* and Ctf19 complex gene knockouts that were not seen in a similar *ipl1-321* screen (Ng *et al.* 2009), we looked in more detail at *iml3*∆ and *chl4*∆. When *iml3*∆ and *chl4*∆ strains were crossed with *ipl1-321*, *ipl1-2*, and *sli15-3* mutants (all in the W303 background), viable double mutants were readily isolated in each case and grew essentially normally at 26°, which is permissive for all three Ts^–^ alleles (Figure 3B). Thus *bir1-17* strains show complete dependence on functional Iml3 and Chl4 in W303, whereas *ipl1-321*, *ipl1-2*, and *sli15-3* strains are virtually independent of Iml3 and Chl4 for normal proliferation. This confirms that at least some of the differences between the genetic interaction profiles of *ipl1-321* and *bir1-17* are genuine and implies that there is a fundamental difference in the way that the *ipl1* and *sli15-3* mutations affect CPC function in comparison with *bir1-17*.

The *sli15*(∆NT) allele removes the first 228 residues of Sli15 and thereby prevents its interaction with Bir1. Surprisingly, this is not lethal as anticipated, despite the delocalization of Ipl1 from kinetochores as a result of the truncation (Campbell and Desai 2013), leading to the idea that while targeting of the CPC to the kinetochore may be important for efficient chromosome biorientation, it is not essential for it to occur. However, the combination of *sli15*(∆NT) with either *ctf19*∆ or *mcm21*∆ was almost lethal (Campbell and Desai 2013). We therefore crossed *sli15*(∆NT) with *iml3*∆ and *chl4*∆ and, although the double mutants could be obtained, in contrast to *sli15-3* (Figure 3B), there was a clear negative genetic interaction in both cases and the *chl4*∆ *sli15*(∆NT) double mutant was inviable at 35°, a temperature at which each single mutant grew normally (Figure 3C). Thus *bir1-17* and *sli15*(∆NT) share a clear negative genetic interaction with loss of Iml3 or Chl4 that is not seen in *ipl1* mutants or *sli15-3*. Double *nbl1-6 chl4*∆ and *nbl1-6 iml3*∆ mutants could also be obtained, but again showed a strong negative genetic interaction at 35° or above (Figure 3C). In summary, mutations that affect the targeting of the CPC [*nbl1-6*, *sli15(*∆*NT)* and *bir1-17*] show strong negative genetic interactions with loss of either *IML3* or *CHL4*, whereas mutations primarily affecting CPC’s protein kinase activity (*ipl1-2*, *ipl1-321*, and *sli15-3*) do not.

### bir1-17 does not reduce accumulation of pericentromeric cohesin

**Figure 4:**
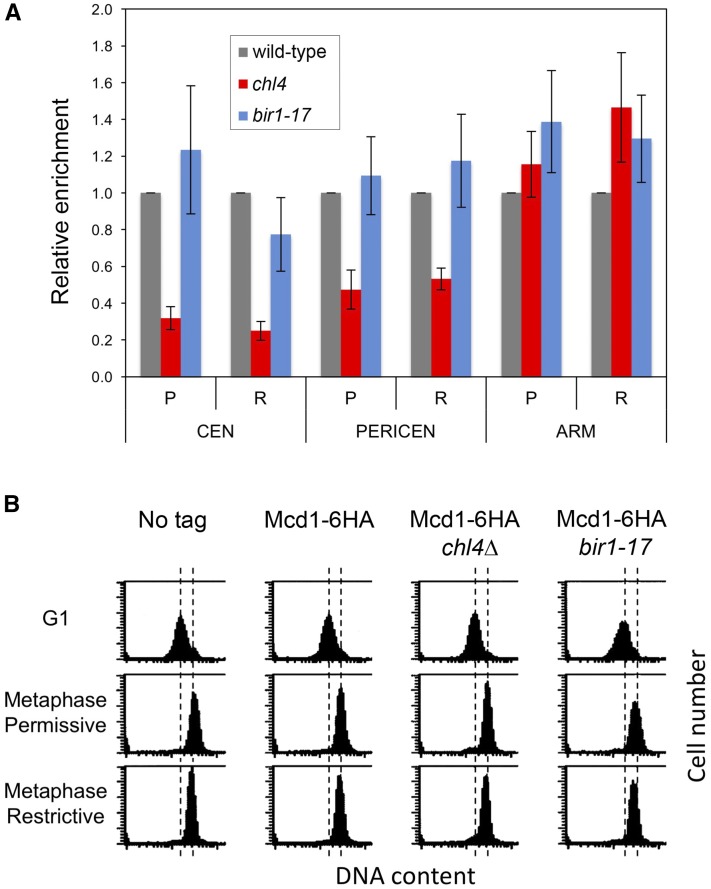
Accumulation of cohesin at the centromere and in the pericentromeric region is not defective in *bir1-17*. Analysis of Mcd1-6HA in wild-type, *bir1-17*, and *chl4*∆ cells, first synchronized in G1 with α-factor at 25° and then released for 3 h at temperatures either permissive (25°; P) or restrictive (37°; R) for *bir1-17* in the presence of nocodazole and benomyl to induce a metaphase arrest. (A) Mcd1 association with the centromeric (CEN), pericentromeric (PERICEN), and arm (ARM) regions of chromosome IV in the two mutant strains relative to the wild-type strain was examined by chromatin immunoprecipitation (ChIP) using an anti-HA antibody. The mean of three independent experiments is shown with error bars indicating the SE. (B) FACS analysis of DNA content confirming synchronization in mitotic metaphase at either temperature.

The Ctf19 kinetochore complex is important for establishment of pericentromeric cohesion, while Csm3 is needed for ensuring that pericentromeric cohesin functions to hold sister centromeres together and shows an additive phenotype with mutations affecting the Ctf19 complex (Fernius and Marston 2009). We therefore examined whether the *bir1-17* mutant might also have a defect in the accumulation of pericentromeric cohesin that could explain its strong negative genetic interactions with knockouts affecting the Ctf19 and *Csm3*-*Mrc1*-*Tof1* complexes. After synchronizing cells in G1 and then releasing them at 37° in the presence of benomyl and nocodazole, association of cohesin at the centromere, pericentromere, and arm regions of chromosome IV was quantified in metaphase-arrested cells by chromatin immune precipitation using HA-tagged Mcd1. Relative to a wild-type control, the *bir1-17* strain showed no obvious defect in the association of cohesin with the centromeric and pericentromeric regions (Figure 4). In contrast, a *chl4*∆ strain showed a clear deficiency in the level of cohesin at the centromere and pericentromere but not in accumulation of cohesin on the chromosome arm, as found previously (Fernius and Marston 2009). Thus defective accumulation of cohesin around the centromere in *bir1-17* is unlikely to provide an explanation for its strong negative interaction with other mutations affecting pericentromeric cohesion. However, we cannot exclude the possibility that, as in *csm3* mutants (Fernius and Marston 2009), the pericentromeric cohesin that accumulates is not fully functional.

### Sgo1 overexpression does not relieve the requirement for Chl4 and Iml3 in bir1-17 strains

Since Iml3 and Chl4 are also needed for pericentromeric accumulation of Sgo1, which is involved both in CPC recruitment and the bias of sister kinetochores to form bioriented attachments (Verzijlbergen *et al.* 2014), we tested whether boosting Sgo1 expression could suppress the synthetic lethality between *iml3*∆ or *chl4*∆ and *bir1-17*. To do this and to provide a platform for further analysis of the defect in the double mutants, we utilized the “anchor away” system, in which proteins that function within the nucleus can be excluded by the addition of rapamycin, thereby triggering conditional loss of function (Haruki *et al.* 2008). This was carried out in the context of a *TOR1-1* background so that cells were resistant to growth inhibition by rapamycin, and rapamycin-induced effects can therefore be ascribed solely to nuclear exclusion of the protein of interest. To verify the use of this approach with the Ctf19 kinetochore complex, we tagged each of the two essential Ctf19 complex members (Ame1 and Okp1) with FRB-GFP and demonstrated that cells could no longer grow when rapamycin was added, while strains in which the nonessential Iml3 and Chl4 were FRB-tagged allowed robust growth on rapamycin (Figure S10A). This analysis also confirmed that nuclear exclusion of Iml3 and Chl4 did not interfere with other essential components of the kinetochore, for example through them “piggy-backing” out of the nucleus with the tagged protein. Microscopy of both the *Iml3*-FRB-GFP and *Chl4*-FRB-GFP strains confirmed that kinetochore localization of either tagged protein was lost within 50 min of rapamycin treatment (Figure S10B).

**Figure 5:**
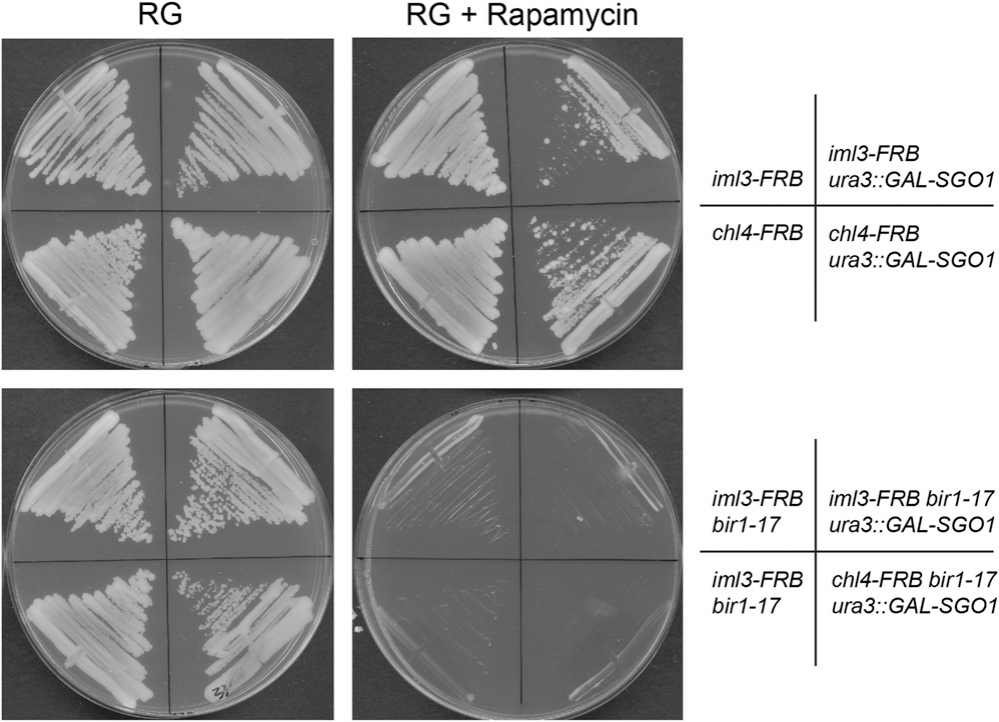
Boosting Sgo1 expression does not overcome the requirement for Chl4 and Iml3 in *bir1-17* strains. Strains with the indicated genotypes (in a *TOR1-1* background) were grown on YPAD medium containing 2% raffinose and 2% galactose to induce expression of *GAL-SGO1* where present, either in the absence (left panels) or presence (right panels) of 10 µg/ml rapamycin to induce nuclear exclusion of Iml3 or Chl4. Plates were photographed after 2 d of growth at 25°. Several other independent isolates of each *ura3*::*GAL-SGO1* strain showed the same properties.

To test whether boosting Sgo1 levels could reverse the synthetic lethality between *bir1-17* and loss of Iml3 or Chl4, *bir1-17* was introduced into the *Iml3*-FRB-GFP and *Chl4*-FRB-GFP strains, and then a galactose-inducible *GAL-SGO1* construct introduced. As shown in Figure 5, addition of rapamycin to the growth medium recapitulated the synthetic lethal phenotype of *bir1-17* with *iml3*∆ or *chl4*∆ gene knockouts. The rapamycin-induced lethality was not overcome by induction of the *SGO1* overexpression construct. Furthermore, inducing *SGO1* expression appeared, if anything, somewhat detrimental to the growth of strains lacking nuclear Iml3 or Chl4. Iml3 and Chl4 both have a specific role in ensuring correct sister chromatid separation in meiosis II that is not necessarily shared with other Ctf19 components (Marston *et al.* 2004), but Figure 5 confirms that the synthetic lethality seen in genetic crosses (Table S9) is also seen in mitotically proliferating cells.

## Discussion

Here, we present the results of a QFA screen using the temperature-sensitive *bir1-17* mutant that is defective in chromosome biorientation to identify both enhancers and suppressors of *bir1-17*. By screening close to the maximum permissive temperature for *bir1-17* at 37°, many strong enhancers and suppressors of *bir1-17* were identified. Furthermore, QFA performed following spotting, was easily able to identify fitness differences between strains that pinning (SGA) could not (see Figure S2). The strong enhancer set contained groups of genes involved in chromosome segregation, sister chromatid cohesion, and microtubule-based processes, consistent with the known function of Bir1 within the CPC, along with additional categories. Our QFA analysis was therefore successful in identifying strong genetic interactions with a subset of genes that, like *BIR1*, are involved in processes related to chromosome segregation. In contrast, only a very small number of enhancers were identified that affected fitness at 20 and/or 27°, temperatures that are fully permissive for *bir1-17*, and overall only four true synthetic lethal interactions were observed (with *kar3*∆, *coq2*∆, *sac1*∆, and *yme1*∆) within the statistically significant strong enhancers that we identified. With the exception of *kar3*∆ (see above), none of these genes show any obvious functional connection with *BIR1*.

The negative genetic interaction profiles of *ipl1-321*, *bir1-17*, and *mcd1-1* showed considerable overlap, consistent with the importance of sister chromatid cohesion for chromosome biorientation, and the *bir1-17* screen highlighted the importance of all the core components of the Ctf19 kinetochore complex in cells where Bir1 function is compromised. All of the interactions between *bir1-17* and Ctf19 complex members and several of the other interactions identified by QFA involving the kinesin Cin8, the Tof1 complex, RFC^Ctf18^, and tRNA wobble uridine modification were independently verified in the W303 background. Despite the expected overlap that we saw between the enhancers of *ipl1-321* and *bir1-17*, given that Ipl1 and Bir1 function together within the CPC, we nonetheless identified several genes, of which *IML3* and *CHL4* are notable examples, that become essential only in *bir1-17* and not in *ipl1-321* and thus support the notion that the *ipl1* and *bir1* mutations affect CPC function in somewhat different ways.

Unlike the enhancers, GO analysis of the *bir1-17* suppressors identified by QFA did not highlight chromosome or microtubule-based functions, but instead identified genes involved in mRNA catabolism (principally the NMD pathway) or genes encoding LRSU proteins as *bir1-17* suppressors. While we cannot exclude the possibility that these suppressors impinge directly on kinetochore function, the CPC or sister chromatid cohesion, we consider it more likely that they relieve the temperature-sensitivity of *bir1-17* by altering the expression level of one or more proteins that are relevant to Bir1 function, and that these suppressors therefore act in a less direct manner. Loss of the NMD pathway and components of the LRSU have been isolated as suppressors in other unrelated SGA screens, emphasizing their potential to act pleiotropically (Addinall *et al.* 2011). These components may therefore play a role in restricting the viability of partial loss of function mutations in a wide range of essential genes, countering the mutational buffering capacity provided by heat shock proteins that allows such mutations to survive (Rutherford 2003).

A single kinetochore protein gene knockout (*ybp2*∆) was identified as a suppressor of *bir1-17*. Ybp2 shows interactions with the COMA and Ndc80 kinetochore subcomplexes and in its absence there are changes in the interactions between members of the KNL [Knl1 (Spc105)-Ndc80-Mis12] network in the kinetochore (Ohkuni *et al.* 2008). This is reminiscent of *cnn1*∆, which we identified as a strong enhancer of *bir1-17*: Cnn1 also interacts with the Ndc80 complex and *cnn1*∆ also affects interactions within the KNM network within the kinetochore (Bock *et al.* 2012). Identification of *CNN1* and *YBP2* as strong genetic interactors of *BIR1* might therefore indicate a role of the CPC in modulating interactions within the KNM network and is consistent with the notion proposed by Bock *et al.* (2012) that Cnn1 and Ybp2 could act in overlapping pathways that regulate KNM interactions during the cell cycle.

Although the Ctf19 complex is a component of the kinetochore, it is now clear that it plays a key role in providing a signal for the deposition of pericentromeric cohesin on chromosomes (Fernius and Marston 2009), which in turn leads to the recruitment of Sgo1 and condensin (Verzijlbergen *et al.* 2014). The pericentromeric region has a specialized structure (Yeh *et al.* 2008) and it is likely that the accumulation of cohesin and condensin in this region is part of a mechanism that provides an intrinsic bias toward bioriented attachment of sister chromatids to spindle microtubules. Iml3 and Chl4 form a heterodimer (Hinshaw and Harrison 2013) that is a peripheral component of the Ctf19 complex, based on the assembly dependencies that have been established for Ctf19 complex (Pot *et al.* 2003). However, loss of Iml3 or Chl4 is sufficient to disrupt the association of cohesin, condensin, and Sgo1 with the pericentromere, despite not affecting the interaction of other Ctf19 complex members with the kinetochore. Why then, should loss of Iml3 and Chl4 only be a problem in combination with *bir1-17* and not *ipl1-321*? In *ipl1-321*, the mutation affects the catalytic subunit of the CPC (Biggins *et al.* 1999) but it can most likely still be targeted to the kinetochore through its interactions with Sli15, Nbl1, and Bir1. While the *bir1-17* mutation does reduce Ipl1-dependent kinase activity, it also causes significant delocalization of Ipl1 from the kinetochore (Makrantoni and Stark 2009), and under circumstances where Ipl1 is delocalized, we now know that the Ctf19 complex becomes essential for viability (Campbell and Desai 2013). Thus, although kinase function is reduced in *ipl1-321*, because it can be targeted to the kinetochore it may be sufficient to overcome loss of any intrinsic bias toward biorientation that requires Iml3 and *Chl4*-dependent accumulation of cohesin, condensin, and Sgo1 at the pericentromere. Conversely, in *bir1-17* strains as in *nbl1-6* strains (Nakajima *et al.* 2009) and *sli15*(∆NT) strains (Campbell and Desai 2013), delocalization of the CPC from kinetochores may make CPC-dependent error correction less efficient, and cells may now rely much more on the intrinsic bias toward biorientation that ultimately relies on Ctf19 complex-dependent events at the pericentromere. Interestingly, the intrinsic bias toward biorienting chromosomes is greater, and hence the need for CPC-mediated error correction much lower, when microtubule attachment occurs after the SPBs have separated (Indjeian and Murray 2007). This feature may explain the negative genetic interactions between *bir1-17* and either *kar3*∆ or *cin8*∆. These two genes encode motor proteins that are involved in spindle pole separation and both knockouts lead to short spindles (Gardner *et al.* 2008; Hoyt *et al.* 1992; Roof *et al.* 1992; Saunders and Hoyt 1992), which may reduce the intrinsic biorientation bias and lead to a much greater requirement for CPC-mediated error correction.

Since *ctf19*∆ mutant kinetochores lack both Iml3 and Chl4 (Pot *et al.* 2003), the synthetic lethality between *ctf19*∆ and *bir1-17* could, in principle, be explained solely on the basis of loss of Chl4 and Iml3, and this could also be the case for some or all of the other deletions of Ctf19 components that share this phenotype. Why then, might loss of some core Ctf19 complex components such as Mcm21 also lead to inviability in the *ipl1-321* mutant? Perhaps absence of components such as Mcm21 leads to a significantly greater loss of the intrinsic bias toward bi-orientation, or alternatively, these inner components of the Ctf19 complex may have additional roles in kinetochore function, as proposed for their higher eukaryotic counterparts (Suzuki *et al.* 2014), which are separate from their requirement to signal cohesin and condensin deposition at the pericentromere and that lead to reduced kinetochore function when they are absent.

Although we can account for the known phenotypes of the *bir1-17* mutation based on Bir1 being a component of the yeast CPC (Makrantoni and Stark 2009), it is possible that some of the genetic interactions we have found might reflect roles of Bir1 that are independent of it being part of the canonical CPC (*i.e.*, the *Ipl1*-*Sli15*-*Nbl1*-*Bir1* complex). It has been reported that a significant fraction of Bir1 is present in a complex with Sli15 (and possibly also Nbl1) that do not contain Ipl1 (Sandall *et al.* 2006; Thomas and Kaplan 2007) and this complex has been implicated both in septin dynamics (Gillis *et al.* 2005; Thomas and Kaplan 2007) and as a tension sensor at the kinetochore (Sandall *et al.* 2006). We could not find any genes annotated in SGD as being involved in septin function among the *bir1-17* enhancers, although five such genes (*DMA1*, *ELM1*, *GIC2*, *RGA1*, and *SPR3*) were identified as weak *bir1-17* suppressors (Table S8 in File S2) and may relate to the Ipl1-independent role in septin behavior proposed for Bir1 (Thomas and Kaplan 2007). If Sli15-Bir1 does constitute some form of tension-sensing linkage as proposed by Sandall *et al.* (2006), then it is also possible that the interactions we find with the Ctf19 complex could, in part, reflect a requirement for pericentromeric cohesion in promoting tension-sensing.

## Supplementary Material

Supplemental material is available online at www.g3journal.org/lookup/suppl/doi:10.1534/g3.117.300089/-/DC1.
