# Supplementary material for: A Functional Link Between Bir1 and the Saccharomyces cerevisiae Ctf19 Kinetochore Complex Revealed Through Quantitative Fitness Analysis
Source: G3 (Bethesda). 2017 Jul 28;7(9):3203–15. doi: 10.1534/g3.117.300089 (PMC5592945; doi:10.1534/g3.117.300089)
Supplement: Supplementary file 1 [file 3203FileS1.pdf]

SUPPLEMENTAL FILES

File S1. List of Supplemental files

File S2. This file contains Tables S2-S8

Table S2. QFA results obtained by screening at 20°C. Parameters were calculated using the QFA R package, as described previously (Addinall et al. 2011) and data ranked in ascending order of GIS i.e. with the strongest negative interactions (i.e. most negative GIS values) listed first. Columns are as follows: ORF, array deletion ORF systematic name; Gene, standard name of gene corresponding to entry in SGD; P, p-value for significance of difference between observed fitness and predicted fitness assuming genetic independence; Q, q-value (FDR corrected p-value), GIS, Genetic Interaction Strength; the remaining columns tabulate the fitness data for each deletion in the *bir1-17* (query) and control experiments (Fitness Summary), the standard error for the fitness values (QuerySE, Control SE), the number of experiments (Query Count, Control Count), significance test used (t-test in all cases) and whether the knockout enhanced (E, negative GIS) or suppressed (GIS positive, S) the fitness of *bir1-17* mutation.

Table S3. QFA results obtained by screening at 27°C. QFA results obtained by screening at 27°C. Data are presented as described in the Table S2 legend.

Table S4. QFA results obtained by screening at 37°C. QFA results obtained by screening at 37°C. Data are presented as described in the Table S2 legend.

Table S5. Summary of *bir1-17* phenotypic enhancers identified by screening at 37°C. All *bir1-17 yfg*Δ interactions with q-values ≤ 0.05 are ranked in ascending order of GIS i.e. with the strongest negative interactions (i.e. most negative GIS values) listed first. Column labels are as in Table S2 but with the addition of an 'Also affected' column that indicates where one gene deletion overlaps to a greater or lesser extent with another gene, which may therefore account for the *bir1-17* interaction observed - for example YGL217C, where the systematic deletion also removes the first ~12% of *KIP3*.

Table S6. Summary of *bir1-17* phenotypic enhancers identified by screening at 27°C. Parameters and data presentation are as in Table S5.

Table S7. Summary of *bir1-17* phenotypic enhancers identified by screening at 37°C. Parameters and data presentation are as for Table S5.

Table S8. Summary of *bir1-17* phenotypic suppressors identified by screening at 37°C. Parameters and data presentation are as for Table S5 except that the data are ranked in *descending* order of GIS i.e. with the strongest positive interactions (largest GIS values) listed first.

File S3. GO terms enriched in genes identified as statistically significant strong *bir1-17* enhancers (defined as GIS ≤ -25.0; Tables S5-S7) were searched for enrichment of GO terms using the GO term finder (Boyle et al. 2004) at SGD (Cherry et al. 2012, queried April 2017) and using the Process, Function and Component ontologies. GOID, numerical descriptor of each GO term within the ontology; GO term,

description of the GO term; Cluster frequency, % of genes in the input list annotated to the particular GO term; Background frequency, % of all *S. cerevisiae* nuclear-encoded ORFs in the systematic deletion collection minus those excluded as described in Materials and Methods (4235 genes) annotated to the GO term; P-value, the probability or chance of seeing at least x number of genes out of the total n genes in the list annotated to a particular GO term, given the proportion of genes in the background set that are annotated to that GO Term; FDR, false discovery rate determined from 50 simulations with random genes; Expected FP, expected false positives; Genes annotated to each GO term that were identified in the query list of *bir1-17* enhancers are shown in the final column. Analysis was performed in April 2017.

File S4. GO terms enriched in genes identified as strong *bir1-17* suppressors at 37°C. Genes identified as statistically significant strong suppressors at 37°C (defined as GIS > 25.0; Table S8) were searched for enrichment of GO terms as described for the enhancers summarized in File S3.

Table S1. List of primers used.

Table S9. Tetrad analysis demonstrating synthetic lethal interactions between *bir1-17* and selected gene knockouts in the W303 background.

Table S10. Genetic interactions between *bir1-17* and selected gene knockouts in the in W303 background.

Figure S1. Diagram representing the doubly-marked *bir1-17* locus used for QFA. The locations of the *Hph*MX and *Nat*MX flanking markers and positions in the *bir1-17* sequences to which the primers used in the construction of the locus (Table S1) bind are indicated. pTEF1, *Ashbya gossypii TEF1* promoter sequence; tTEF1, *Ashbya gossypii TEF1* terminator sequence.

Figure S2. *The bir1-17* fitness defect can be seen after spotting but not after pinning. (A) Technical replicates of *bir1-17* and wild-type cells were grown after pinning onto a solid agar surface in 1536 format to simulate the final stage of an SGA experiment. Plates were repeatedly photographed during growth. No difference in pinned colony size with genotype is apparent at any temperature tested. (B) *bir1-17* and wild-type cells were taken from the plates shown in panel A, inoculated into liquid culture, grown to saturation and diluted ~1:100 in water before spotting onto a solid agar surface, as during QFA. The temperature sensitivity of *bir1-17* cells is clear at temperatures of 37°C and above (spotted *bir1-17* cultures appear more slowly and are less dense at the end of the experiment).

Figure S3. Fitness plots of *bir1-17* double mutants screened at (A) 20°C and (B) 27°C. Following four replicate crosses of *bir1-17* with the yeast genome knockout collection, Quantitative Fitness Analysis of each *bir1-17 yfg*Δ (“*your favorite gene deletion*”) strain was carried out and mean fitness plotted against the mean fitness observed from 8 replicates of a control cross between a *ura3*Δ strain and the knockout collection. Gene deletions showing a significant positive (blue) or negative (red) genetic interaction with *bir1-17* are indicated, with all other values indicated in grey. A significant interaction was defined as one with a q-value (FDR-corrected p-

value: see Addinall et al. 2011) < 0.05. The line of equal growth (grey dashed) and a population model of expected fitness (solid grey; a regression line based on all the data points) are also indicated. The blue lines show the average position of *his3*Δ strains as a proxy for wild-type growth.

Figure S4. Fitness plot of *bir1-17* double mutants screened at 37°C mapping the positions of all significant genetic interactions. Generation and annotation of the fitness plot was as described in the legend to Figure 1, with the addition that all statistically significant strong suppressors (GIS ≥ 25, q-value ≤ 0.05) were highlighted as purple dots above the regression line (solid grey) and all statistically significant enhancers (GIS ≤ 25, q-value ≤ 0.05) were highlighted as purple dots below the regression line.

Figure S5. Fitness plot of *bir1-17* double mutants highlighting genetic interactions with the Ctf19 kinetochore complex. Generation and annotation of the fitness plot was as described in the legend to Figure 1, with the addition that all double *bir1-17 yfg*Δ mutants where *yfg*Δ affects a member of the Ctf19 kinetochore complex have been highlighted as purple dots. Left panel, fitness plots from screening at 20°C; centre panel, fitness plot from screening at 27°C; right panel, fitness plot from screening at 37°C.

Figure S6. Fitness plot of *bir1-17* double mutants screened at 37°C mapping the positions of genetic interactions falling within six categories. Generation and annotation of the fitness plot was as described in the legend to Figure 1, with the addition that all double *bir1-17 yfg*Δ mutants where *yfg*Δ affects a member of the

indicated group of genes have been highlighted as purple dots. Since all essential genes and a small number of nonessential genes are absent from the systematic deletion collection not all the genes in each group are annotated on the plots. (A) Yeast kinesins (*KIP1*, *KIP2*, *KIP3*, *YGL217W* (overlaps *KIP3*), *CIN8*, *KAR3*, *CIK1*, *VIK1*, *CIN1, BIK1.* (B) Genes related to sister chromatid cohesion and replication: Tof1 complex (*TOF1*, *CSM3*, *MRC1*, *::MRC1*), RFC^{Ctf18} (*CTF8*, *CTF18*, *DCC1*; *RFC2-5* are essential genes), RFC^{RAD24} (RAD24; RFC2-5 are essential genes), PCNA-like clamp (*RAD17*, *DDC1*; *MEC3* not in collection); RFC^{ELG1} (*ELG1*; *RFC2-5* are essential genes), DNA polymerase epsilon (*DPB3*, *DPB4*, *YBR277C* (*::DPB3*); *DBP2* is essential). (C) Histone exchange: *ASF1, SWC5*, *SWR1*, *VPS71*, *VPS72* and *YAF9* (*INO80*, *RVB1*, *RVB2*, *SAS2*, *SWC3, SWC4* and *TUP1* are not in the collection). (D) Chromatin modification: ADA complex (*AHC1*, *AHC2*; *ADA2*, *GCN5* and *NGG1* are not in the collection); Compass (*BRE2*, *SDC1*, *SHG1*, *SPP1*, *SWD1*, *SWD3*), Rpd3S (*EAF3*, *RCO1*, *YMR075C-A* (*::RCO1*), *RPD3*, *SIN3*, *UME1*), Set3C (*CPR1*, *HOS1*, *HST1*, *SET3*, *SET4*, *SIF2*, *SNT1*), ISW (*IOC2*, *IOC4*, *ISW1*, *VPS1*; *MOT1* is not in the collection). (E) tRNA wobble uridine modification (*ATS1*, *ELP2*, *ELP3*, *ELP4*, *ELP6*, *IKI3*, *KTI12*, *NCS2*, *YNL120C* (*::NCS2*), *NCS6*, *SAP185*, *SAP190*, *TUM1*, *UBA4, URM1*; *ELP5* and *DPH3* not in collection). (F) Peroxisomal genes: all double *bir1-17 yfg*Δ mutants where YFG is annotated in SGD as being required for peroxisomal function and the knockout is in the collection (*DJP1*, *FIS1*, *INP1*, *LPX1*, *OAF1*, *PEX1*, *PEX10*, *PEX11*, *PEX12*, *PEX13*, *PEX14*, *PEX15*, *PEX17*, *PEX18*, *PEX19*, *PEX2*, *PEX21*, *PEX22*, *PEX25*, *PEX27*, *PEX28*, *PEX29*, *PEX3*, *PEX30*, *PEX31*, *PEX32*, *PEX34*, *PEX4*, *PEX5*, *PEX6*, *PEX7*, *PEX8*, *PEX9*, *YLL054C*, *YMR018W; PEX35* not in collection).

Figure S7. Fitness plot of *bir1-17* double mutants screened at 37°C mapping the positions of genes involved in ribosome biogenesis and function. Generation and annotation of the fitness plot was as described in the legend to Figure 1, with the addition that the plot was labeled with either (A) all *RPL* genes (encoding components of the large ribosomal subunit: LRSU); (B) all *RPS* genes (encoding components of the small ribosomal subunit); (C) all genes encoding LRSU components or factors involved in the assembly and processing of the LRSU, as defined by their annotation in SGD to GO entries 0042273, 0000027, 0000463, 0000470, 0022625, 0015934 and 0030684 but with any clearly incorrectly annotated genes (e.g. encoding mitochondrial ribosomal proteins) removed manually. Labelled genes are indicated by a purple dot in each case. Note that not all genes defined as indicated are present in the knockout collection.

Figure S8. Fitness plot of *bir1-17* double mutants screened at 37°C mapping the positions of genes involved in (A) mRNA turnover or (B) the COP9 signalosome. Generation and annotation of the fitness plot was as described in the legend to Figure 1, with the addition that the plot was labeled with knockout of genes encoding either (A) mRNA turnover components (all nonessential components of the NMD pathway, SKI complex and exosome as annotated in SGD) or (B) COP9 signalosome components (see Benschop et al. 2010).

Figure S9. Relative fitness of selected gene knockouts alone or in combination with *bir1-17* in the W303 background. Where double *bir1-17 yfg*Δ mutants were viable, the single and double mutants were grown overnight at 26°C in YPAD medium along with a control wild-type strain (K699), adjusted to 1.0 OD_{600} and then spotted out

along with four 10-fold dilutions on YPAD medium and grown at 26°C, 35°C and 37°C for 2 days before imaging. The *irc15*Δ construct, made using primers #736 + #746, removed codons 1-332 of the 499-codon *IRC15* ORF. A construct equivalent to that in the knockout collection (made using primers #746 +#780) that also removed the last eight sense codons of *CTF19* was synthetic lethal with *bir1-17* (not shown). *sin3*Δ was included because it was a strong enhancer at the two lower temperatures, although at 37°C fell just outside out GIS ≤ -25 cut-off for defining strong enhancers.

Figure S10. The ‘Anchor-away’ system can be used to inactivate kinetochore protein function. (A) Growth of rapamycin-resistant *TOR1-1* ‘anchor-away’ strains containing FRB-tagged Iml3, Chl4, Ame1 or Okp1 on rapamycin-containing medium with K699 shown as a *TOR1* rapamycin-sensitive control. Nuclear exclusion of the essential Ame1 and Okp1 kinetochore proteins prevents proliferation, whereas nuclear exclusion of the non-essential Iml3 or Chl4 does not. (B) Visualization of Iml3-FRB and Chl4-FRB by virtue of GFP fluorescence (the FRB tag includes GFP). Left panels, asynchronous cells grown in the absence of rapamycin, with one or two foci present according to cell cycle stage as expected for a kinetochore-localized protein. Right panels, diffuse cellular fluorescence 50 minutes after adding 1 µg/ml rapamycin.
