# Supplementary figures and images for: A Functional Link Between Bir1 and the Saccharomyces cerevisiae Ctf19 Kinetochore Complex Revealed Through Quantitative Fitness Analysis
Source: G3 (Bethesda). 2017 Jul 28;7(9):3203–15. doi: 10.1534/g3.117.300089 (PMC5592945; doi:10.1534/g3.117.300089)

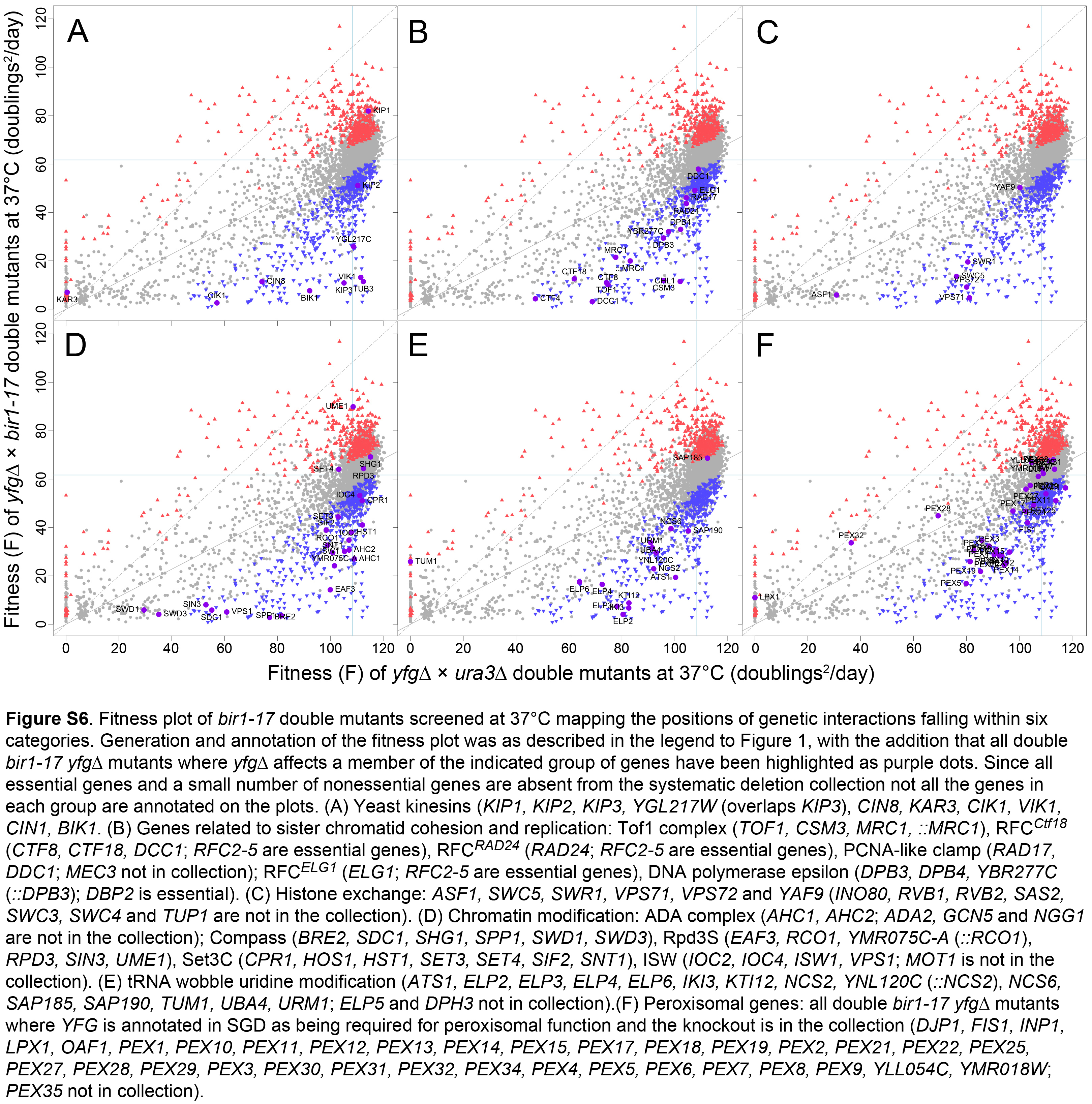

Supplement: Supplementary file 5 [file 3203FigureS6.jpg]
